# Non-ketotic Hyperglycemia Chorea-Ballismus and Intracerebral Hemorrhage: A Case Report and Literature Review

**DOI:** 10.3389/fnins.2021.690761

**Published:** 2021-06-23

**Authors:** Mingming Dong, Jian-Yu E, Liyang Zhang, Weiyu Teng, Li Tian

**Affiliations:** ^1^Department of Neurology, The Fourth People's Hospital of Shenyang, Shenyang, China; ^2^Department of Neurology, The First Hospital of China Medical University, Shenyang, China; ^3^Wilmer Eye Institute, Johns Hopkins University School of Medicine, Baltimore, MD, United States; ^4^Department of Geriatrics, Shengjing Hospital of China Medical University, Shenyang, China

**Keywords:** non-ketotic hyperglycemia chorea-ballismus, diabetic striatopathy, intracerebral hemorrhage, case report, metabolical syndrome, hyperdense signs on neuroimaging, differential diagnosis

## Abstract

Non-ketotic hyperglycemia chorea-ballismus (NKH-CB) is a rare metabolical syndrome secondary to the hyperglycemic condition, which is characterized by a triad of acute or subacute hemichorea-hemiballismus, hyperglycemic state, and unique abnormalities limited to the striatum on neuroimaging. Several related case studies on this disorder have been reported previously, but NKH-CB had never been associated with intracerebral hemorrhage (ICH). Herein, we report an uncommon case of NKH-CB and ICH that occurred simultaneously in one patient, which provides a challenge for clinicians in making a correct diagnosis. An 88-year-old woman with a long-term history of poor-controlled type 2 diabetes mellitus and hypertension, who presented with a sudden-onset headache, restlessness, severe bilateral choreiform and ballistic movements, elevated levels of glucose and osmolality in the serum, an increased white blood cell count, and two-type hyperdense signs on CT imaging, was finally diagnosed with NKH-CB and ICH. Despite administrated active treatments, the patient's clinical status did not improve and ultimately passed away. This case is reported to remind clinicians to consider the possibility of NKH-CB when patients present sudden-onset choreiform and ballistic movements. It is also the first entity with two-type hyperdense signs on CT imaging simultaneously, which helps us distinguish NKH-CB from ICH more intuitively.

## Introduction

Non-ketotic hyperglycemia chorea-ballismus (NKH-CB) is a rare metabolical syndrome secondary to the hyperglycemic condition. The classic clinical manifestation of this disorder includes a triad of acute or subacute hemichorea-hemiballismus, hyperglycemic state, and unique reversible abnormalities limited to the striatum on neuroimaging (Wintermark et al., 2004). Because of unfamiliarity and radiographic features easily misdiagnosed as intracerebral hemorrhage (ICH) for most physicians, the prevalence of NKH-CB is believed to be underestimated with the rate of ~1 in 100,000 (Ondo, 2011). This disorder has been reported to occur predominantly in elderly Asian women with type 2 diabetes mellitus and generally has a favorable prognosis after correcting hyperglycemia. Currently, several related case studies on this disorder have been reported previously, but NKH-CB had never been associated with ICH. Herein, we describe an uncommon case of NKH-CB and ICH that occurred simultaneously in one patient, which provides a challenge for clinicians in making a correct diagnosis. Despite administrated active treatments, the patient's clinical status did not improve and ultimately died. We report this case to remind clinicians to be familiar with and to recognize early this unusual disorder. We also discuss potential mechanisms that lead to a poor prognosis of our case.

## Case Report

An 88-year-old woman with a long-term history of poor-controlled type 2 diabetes mellitus and hypertension was admitted to the emergency department for sudden onset of headache, restlessness, and severe involuntary movements of the four limbs for 1 day. During transit to our hospital, she became speech confused after vomiting blood. The patient's family denied that she had family history, drug and toxic exposure, which led to abnormal movement.

The patient had no fever and normal vital signs except for blood pressure 184/98 mmHg. On examination, she was restless, nonsense, confused, and unable to converse correctly. Bilateral pupils were normal and reactive to light with no Kayser–Fleischer ring. She presented with remarkable bilateral choreiform and ballistic movements of the limbs, which were more severe in the upper limbs (continuous, irregular, and involuntary jerking movements) with hypotonia and normal muscle strength. The tendon reflex, neck resistance, and Babinski sign were negative. She could not cooperate with the remaining examinations (other cranial nerves, sensation, coordination, and gait).

**Figure 1:**
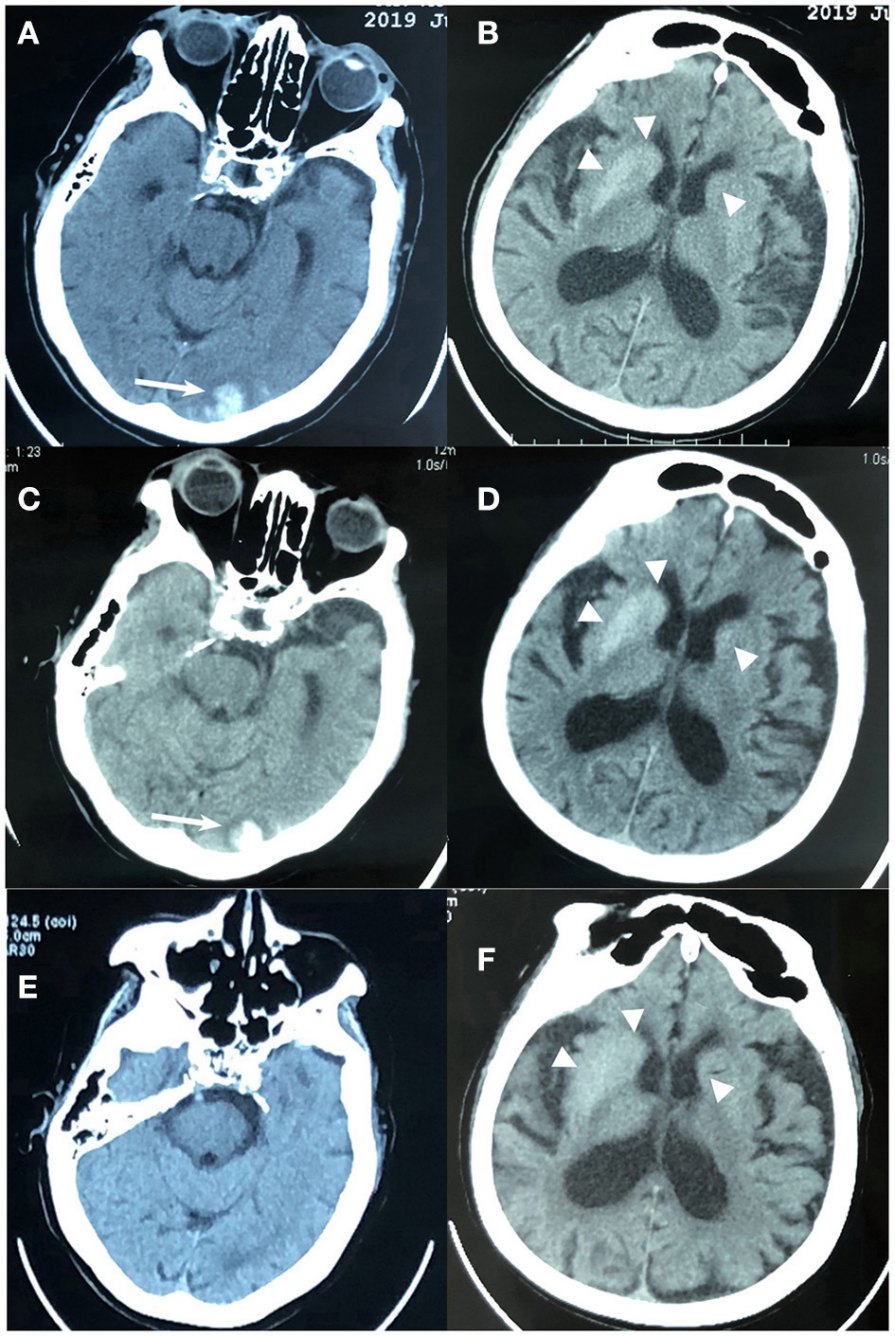
Brain computed tomography (CT) imaging of an 88-year-old woman with non-ketotic hyperglycemia chorea-ballism and intracerebral hemorrhage. **(A,B)** At admission, CT showed hyperdense lesions in the left occipital lobe (white arrow), right caudate nucleus and putamen (triangle), and left caudate (triangle). **(C,D)** Eight hours later after admission, CT showed mild hematoma enlargement, slightly increased edema around the occipital lobe lesion (white arrow), and no significant changes in the bilateral striatum regions (triangle); **(E,F)** 15 days before admission, CT showed hyperdensity in bilateral striatum regions (triangle) without edema and occipital lobe lesion.

Laboratory results on the emergency (Table 1, first day) were clinically significant for leukocytosis, neutrophilia, elevated C-reactive protein, hyperglycemia, elevated serum osmolality, positive urine sugar, negative urine ketone, and hypoxemia. A non-contrast brain computed tomography (CT) showed hyperdensity in the left occipital lobe with a mean CT attenuation value of 72 Hounsfield unit (HU) and bilateral striatal regions (right caudate nucleus and putamen with a mean value of 49 HU, left caudate nucleus with a mean value of 43 HU) (Figures 1A,B).

**Table 1:** Laboratory investigations on admission (first day) and sixth day in hospital.

**Investigation**	**Result**		**Reference range**
	**First day**	**Sixth day**	
White blood cell count, × 10^9^/L	10.04	14.21	3.5–9.5
Absolute neutrophil count, × 10^9^/L	9.07	13.15	1.8–6.3
Absolute lymphocyte count, × 10^9^/L	1.2	0.5	1.1–3.2
C-reactive protein, mg/L	4.34	179.73	0.1–10.0
Serum glucose, mg/dl	300.6	599.4	70.2–109.8
Hemoglobin A1c, %	15.2	NA	3.8–6.5
Urea nitrogen, mmol/L	6.66	27.45	3.1–8.8
Creatinine, μmol/L	44	187	41–81
Sodium, mmol/L	141	133	137–147
Potassium, mmol/L	4.5	4.57	3.50–5.30
Serum osmolality, mOsm/kg	314.36	335.89	280–310
Arterial blood gas			
pH	7.333	NA	7.350–7.450
PaO_2_, mmHg	57.5	NA	83.0–108.0
PaCO_2_, mmHg	23.7	NA	32.0–48.0
HCO${}_{3}^{-}$, mmol/L	15.5	NA	22.0–27.8
Urine glucose	4+	NA	–
Urine ketone	–	NA	–

*NA, not applicable*.

The emergency physicians considered those lesions as acute ICH for the first time, and the patient was then admitted to our neurology department. A contrasted brain CT at 8 h later of hospitalization, however, disclosed the over-time evolution differences in these lesions: slightly increased edema around the occipital lobe lesion with mild hematoma enlargement (a mean value of 75 HU), whereas no significant changes in the striatal regions were observed (still regular, well-defined, limited without edema, and mass effect; a mean value of 51 HU and 44 HU in the right and left side, respectively), reassuring that the striatum lesions were not hemorrhagic damage (Figures 1C,D). Supplemental laboratory examinations including thyroid and parathyroid function, serum electrolyte levels, coagulation profile, autoimmune antibodies, liver and kidney function, ceruloplasmin, vitamins D and B12, folic acid, human immunodeficiency virus antibody, syphilis antibody, erythrocyte sedimentation rate, anti-O antibody, and serum cancer markers were normal. The chest X-ray indicated inflammation in the upper-lobe segment of the left lung. Based on these clinical data and excluding the possibility of other diseases, the patient was ultimately diagnosed with NKH-CB and ICH. The secondary diagnoses included stress-related upper gastrointestinal bleeding, aspiration pneumonia, and hypertension. A chart review of her 15-day-ago records with a CT image of hyperdensity in bilateral striatum (a mean value of 46 HU and 43 HU in the right and left side, respectively) without edema, mass effect, and occipital lobe lesion (Figures 1E,F) further confirmed our diagnosis. At that time, the patient only complained of mild dizziness for 3 days; however, no subsequent investigations were conducted because the then clinician paid insufficient attention to these abnormal neuroimaging findings, as well as considered that the symptom of the patient was mild.

The patient received supportive management, including the use of a cardiac monitor, oxygen, anti-hyperglycemic therapy with fluids and insulin, as well as a series of symptomatic treatments, including controlling choreiform and ballistic movements with clonazepam (1 mg per night), anti-hypertensive, anti-inflammatory, and acid suppression therapy. On the third day, with the reduction of hyperglycemia (blood glucose was controlled at 163.8–189 mg/dl), the symptoms of chorea-ballismus and confusion improved, and the patient could converse briefly. The patient denied any visual symptoms such as blurred and distorted vision. Three days later, the patient had a fever (38.0°C) with the laboratory results worsening (Table 1, sixth day), and her condition deteriorated rapidly to coma without chorea-ballismus movements due to severe uncontrolled infection and recurrent hyperglycemic crisis. Despite administrated intensive treatments, including fluid resuscitation, glycemic control, and anti-inflammation, the patient's clinical status did not improve and her relatives decided to suspend treatments due to limited financial support. On day 8, unfortunately, the patient developed digestive tract rebleeding and ultimately passed away.

## Discussion

KH-CB, also recognized as diabetic striatopathy, is a rare complication of diabetes mellitus (Xiao et al., 2019) and was first described by Bedwell (1960). Chua et al. (2020) found that NKH-CB frequently occurs in Asia (71.6% in total reported cases), particularly the older women with new-onset or uncontrolled diabetes, with an average onset age of 67.6 years old, and the sex ratio (male to female) was 1:1.7. Among the numerous causes of chorea-ballismus, non-ketotic hyperglycemia is the most common in the metabolic cause group, but it is still scarce with roughly 1% of the total etiologies in a recent study by Mayo Clinic (Ryan et al., 2018). Besides, the chorea-ballismus movement can also be observed in some ketotic diabetic patients, adolescents with newly diagnosed diabetes, and even children (Tan Y. et al., 2014; Aquino et al., 2015; Faundez et al., 2016). Other causes that could induce acute/subacute-onset choreiform movement must be considered (Table 2).

**Table 2:** Causes of acute/subacute-onset choreiform movement.

**Causes**	**Vascular**	**Metabolic**	**Infectious**	**Immune-mediated**	**Drug-induced**	**Others**
Disorders	Ischemic/hemorrhagic stroke	Hypoglycemia/hyperglycemia (non-ketotic)	Viral encephalitis	Rheumatologic diseases (SLE, APS, SS)	Neuroleptics Levodopa, dopamine agonists	Pregnancy Polycythemia vera
	Vascular malformation	Electrolyte imbalance (hyponatremia/hypernatremia, hypercalcemia, hypomagnesemia)	Group A beta-hemolytic streptococcus (SC)	Autoimmune neurologic syndromes/ paraneoplastic syndromes	Antiepileptic drugs (phenytoin, carbamazepine, valproic acid)	Tumors Carbon monoxide intoxication
		Hyperthyroidism, hypoparathyroidism/ hyperparathyroidism	Parasitic (toxoplasmosis, cysticercosis)	Demyelinating disease	Psychostimulants (cocaine, amphetamines)	Psychogenic chorea
		Acquired hepatolenticular degeneration	Cryptococcal granuloma Mycoplasma Tuberculoma Neurosyphilis		Lithium Oral contraceptives, estrogen replacement therapy Steroids	
			HIV encephalitis Prion diseases		Methotrexate, cyclosporine Fluoroquinolones	

*SC, Sydenham's chorea; HIV, human immunodeficiency virus; SLE, systemic lupus erythematosus; APS, antiphospholipid antibody syndrome; SS, Sjögren syndrome*.

Chorea is hyperkinetic dyskinesia characterized as being involuntary, irregular, unpredictable, small-amplitude, and more distal, whereas ballismus is featured as being large-amplitude, arrhythmic, and more proximal (especially at the shoulder and hip). The ballismus often evolves into chorea. These involuntary movements generally develop while awake, become violent in an emotional mood, and disappear during sleep. Chorea-ballismus can appear in the large majority of patients with diabetic striatopathy (97.7%) (Chua et al., 2020). The unilateral upper and lower limbs are most frequently affected, but rare bilaterally (only 9.7%) (Oh et al., 2002; Chua et al., 2020). Also, the facial and tongue muscle can be involved, which could present with eyebrow extrusion, mouth skimming, tongue extension, and other symptoms. The movement disorder usually occurs with severe hyperglycemia simultaneously and is also ameliorated with the recovery of blood glycemic levels. Therefore, the prognosis is generally well after correcting hyperglycemia. In the present case, the patient presented with rare bilateral limb involvements, and after reduction of hyperglycemia, her symptoms were also once relieved.

The laboratory test is essential for screening hyperglycemic status. A previous meta-analysis showed that on the onset day of NKH-CB, the mean serum glycemic level was 481.5 (range, 169–1,264) mg/dl, HbA1c level was 14.4% (range, 9.9–19.2%), and serum osmolarity was 305.9 (range, 291–335) mmol/kg (Oh et al., 2002). Chua et al. (2020) updated the data of average serum glycemic level (414 mg/dl) and HbA1c level (13.1%).

The features of neuroimaging are the reversible hyperdense and hyperintense lesions limited to the contralateral striatum areas of the affected limbs without edema and mass effect, on CT and T1-weighted sequences magnetic resonance imaging (MRI), respectively (Oh et al., 2002). The radiological findings can be variable signals on T2-weighted, diffusion-weighted imaging, and fluid-attenuation inversion recovery sequences of MRI, and no enhancement on an enhanced scan (Chu et al., 2002; Oh et al., 2002; Zheng et al., 2020). Despite the highly significant correlation between CT and MRI findings, the recent study has shown that there is about one-sixth mismatch and incompatibility between CT and MRI results. MRI is more sensitive than CT in detecting striatal abnormalities associated with diabetic striatopathy (Chua et al., 2020). Additionally, there were a few reported cases with negative findings on both CT and MRI (Chang et al., 2017). Of the three striatal regions, the putamen is the most commonly involved nucleus, and it can be affected isolatedly or in combination with the caudate nucleus and/or globus pallidus (Oh et al., 2002). Similarly, bilateral striatum involvement was uncommon (9.7%), consistent with bilateral symptoms. Abnormal imaging performances can last for several months, even years after blood glucose correction (Oh et al., 2002).

In the context of association with ICH, NKH-CB could be more easily misdiagnosed as ICH due to the hyperdense on CT imaging initially. The different evolution of lesions between ICH and NKH-CB on a contrasted CT could aid in the differential diagnosis. Perihematoedema, a prominent pathological feature, and corresponding to the hypodense on CT scan that surrounds intracerebral hematomas, can appear in the early-stage acute ICH and its volume starts to increase 1 h after the onset and grow significantly in the first 24 h (Wagner et al., 1996; Gebel et al., 2002). Moreover, in the acute stage of ICH, the hematoma's CT-attenuation value can rapidly increase to 60–80 HU, even to 80–100 HU in the maturation of hematoma, which is generally attributed to the globin (protein) component of the hemoglobin in a hematoma (Brooks et al., 1989; Parizel et al., 2001). However, these developments following hematoma on CT imaging cannot be observed in NKH-CB. In the present case, MRI was not obtained from this patient due to the severe clinical condition, but through analyzing the over-time developments on CT scan, diabetic history, and a series of laboratory results, the diagnosis of NKH-CB was finally established. Some histological studies have found that the hyperdense striatal lesions were usually characterized by gliosis, selective neuronal loss, and reactive astrocytes rather than the evidence of hemorrhage or infarction within these areas (Shan et al., 1998; Lee et al., 2002; Cherian et al., 2009). Of course, if conditions permit, MRI remains an essential method helpful in not only diagnosis but also judging prognosis (by the value of apparent diffusion coefficient maps) (Chu et al., 2002; Zheng et al., 2020). Selected other causes of hyperdense/hyperintense striatal region on CT or T1-weighted MRI should be also considered for the initial visit (Table 3). Our case is the first entity with two-type hyperdense signs simultaneously, which helps us distinguish NKH-CB from ICH through the perspective of neuroimaging more intuitively.

**Table 3:** Other causes of hyperdense/hyperintense striatal region on CT and T1-weighted MRI.

**Imaging features**	**Disorder**	**Regions of involvement**
Hyperdense on CT	Physiologic calcification	Bilateral GP, epiphysis, choroid plexus, habenular nucleus, cerebral falx
	Pathological calcification (Fahr's disease, parathyroid dysfunction)	Symmetrically bilateral CN, PN, GP, thalamus, dentate nuclei, and subcortical white matter
Hyperintense on T1-weighted MRI	Liver disease	Symmetrically bilateral GP; Substantia nigra
	Neurofibromatosis type 1	Bilateral GP
	Carbon monoxide	Bilateral GP; delayed leukoencephalopathy
	Parenteral nutrition	Symmetrically bilateral GP and thalamus

*CT, computed tomography, MRI, magnetic resonance imaging, GP, globus pallidus, CN, caudate nucleus, PN, putamen nucleus*.

Once a diagnosis of NKH-CB is established, prompt correction of blood glucose with fluids and insulin and anti-chorea treatment is recommended. About 26% of patients can reach a benign outcome using glucose-control-only medicine, but additional anti-chorea medications can increase the rate up to 76% (Chua et al., 2020). The most frequent medication used for chorea treatment is haloperidol with high effectiveness, followed by clonazepam, tetrabenazine, and tiapride (Chua et al., 2020). Besides that, surgical interventions, such as thalamotomy and deep brain stimulation, are also effectively adopted in some refractory cases (Takamatsu et al., 1995; Nakano et al., 2005; Goto et al., 2010). Given the potential adverse side effects of haloperidol, such as metabolic syndrome, dysphoria, and even increased short-term mortality risk of 107% (especially in the elderly), we did not use this high potent but high-risk regime for our advanced-age critically ill patient (Schneeweiss et al., 2007; Huybrechts et al., 2012; Tyler et al., 2017; Ventriglio et al., 2018). Instead, we chose clonazepam (a benzodiazepine) because clinical evidence supports that antichorea drug is relatively safe (Peiris et al., 1976; Patorno et al., 2017).

Unlike previously reported cases with a favorable prognosis, our case was a critically ill patient accompanied by multiple complications that result in a poor prognosis. The exact pathophysiological mechanism of NKH-CB remains unclear currently, but several hypotheses have been proposed. Given these hypotheses, the interaction of multiple possible mechanisms might explain our case: (1) in a hyperglycemic state, the dysfunction of the striatum could be induced and even aggravated by a series of pathophysiological reactions including anaerobic metabolism of brain cells caused by hypoperfusion and glycometabolism failure, gamma-aminobutyric acid (GABA) depletion of anaerobic metabolism in the striatum due to the non-ketotic state, ischemia of striatal neurons resulting from destroyed blood–brain barrier caused by blood hyperviscosity, and hypersensitivity of dopamine system receptors secondary to the decreased concentration of estrogen in postmenopausal women (Nath et al., 2006; Cheema et al., 2011; Slabu et al., 2011; Tocco et al., 2016). The synergistic effects of imbalanced dopamine and GABA systems and vascular insufficiency may further exacerbate the striatum's dysfunction, which leads to movement disorders (Lin, 2001). (2) In the condition of uncontrolled diabetes, acute ICH could aggravate hyperglycemia by acute stress, metabolic alteration, and autonomic hormonal changes (Xi et al., 2006; Guo et al., 2016). In that situation, hyperglycemia could become uneasy to control due to various secondary responses, such as interference with insulin-mediated glucose uptake induced by increased epinephrine, and insulin resistance (Halter et al., 1984; Tan X. et al., 2014). Moreover, inflammation can markedly enhance insulin resistance, aggravate stress response, and ultimately lead to a deteriorated hyperglycemic state causing rebleeding from a well-controlled hemorrhagic disorder and curing hardly (McAlister et al., 2005; Dungan et al., 2009). Both hyperglycemia and aspiration pneumonia can significantly increase the risks of in-hospital complications and death (Rueda et al., 2010; Mandell and Niederman, 2019). A vicious cycle composed of hyperglycemia, severe infection, and hemorrhagic disease ultimately led to the unfortunate outcome of our case. Of note, in the recurrent hyperglycemic crisis, we did not observe a return of chorea-ballismus movements for this patient, which might result from the hyperosmolar coma caused by acute deterioration of blood glucose status. In addition, another hyperglycemic crisis could produce a further decrease of cerebral blood flow in the striatal areas, even resulting in the striatal ischemic damage that may interrupt the hyperactivity of the basal ganglia motor circuit (Guisado and Arieff, 1975; Kim et al., 2011).

## Limitations

Since there was no history of prodromal infections as well as evidence of autoimmune-related diseases, and considering that the infection was caused by aspiration pneumonia, the cerebrospinal fluid (CSF) testing was absent. However, the CSF test can improve the level of diagnostic certainty and be helpful for the exclusion of several other diseases. Moreover, the autopsy pathological study was not available due to the lack of informed consent from her family. The pathological study of the striatum may further specifically explain the nature of the striatal lesions and the pathophysiological mechanism of NKH-CB.

## Conclusion

NKH-CB is an uncommon metabolic syndrome and generally has a good prognosis. When clinicians are faced with acute or subacute choreiform and ballistic movements, the possibility of NKH-CB should be considered, while serum glucose levels, serum osmolality, and HbA1c levels should be considered as routine tests. Due to the hyperdense sign on CT imaging, sudden-onset NKH-CB can be easily misdiagnosed, particularly in the setting of complicated ICH. In the context of absent MRI, dynamic observation of CT imaging evolutions aids for differential diagnosis, particularly CT-attenuation values. Although the prognosis of most patients with NKH-CB is usually good, hemorrhagic disease (ICH and stress-related upper gastrointestinal bleeding) and infection may be risk factors that exacerbate the condition.

## Data Availability Statement

The original contributions presented in the study are included in the article/Supplementary Material, further inquiries can be directed to the corresponding author/s.

## Ethics Statement

Written informed consent was obtained from the individual(s) for the publication of any potentially identifiable images or data included in this article.

## Author Contributions

WT and LT contributed to the conception and design of the manuscript. MD and LZ collected the data and drafted the manuscript. J-YE reviewed and modified the manuscript. All authors contributed to manuscript revision, read, and approved the submitted version.

## Conflict of Interest

The authors declare that the research was conducted in the absence of any commercial or financial relationships that could be construed as a potential conflict of interest.
