# Supplementary material for: Non-ketotic Hyperglycemia Chorea-Ballismus and Intracerebral Hemorrhage: A Case Report and Literature Review
Source: Front Neurosci. 2021 Jun 23;15:690761. doi: 10.3389/fnins.2021.690761 (PMC8260933; doi:10.3389/fnins.2021.690761)
Supplement: Supplementary file 1 [file Data_Sheet_1.PDF]

CARE
case report guidelines

CARE Checklist of information to include when writing a case report

(cc) BY-NC-ND

Topic	Item	Checklist item description	Reported on Line
Title	1	The diagnosis or intervention of primary focus followed by the words "case report"	Page 1, line 1
Key Words	2	2 to 5 key words that identify diagnoses or interventions in this case report, including "case report"	Page 1, line 17
Abstract (no references)	3a	Introduction: What is unique about this case and what does it add to the scientific literature?	Page 1, line 21 and 32
	3b	Main symptoms and/or important clinical findings	Page 1, line 27
	3c	The main diagnoses, therapeutic interventions, and outcomes	Page 1, line 31
	3d	Conclusion—What is the main "take-away" lesson(s) from this case?	Page 1, line 32
Introduction	4	One or two paragraphs summarizing why this case is unique (**may include references**)	Page 2, line 37
Patient Information	5a	De-identified patient specific information.	Page 2, line 53
	5b	Primary concerns and symptoms of the patient.	Page 2, line 54
	5c	Medical, family, and psycho-social history including relevant genetic information	Page 2, line 56
	5d	Relevant past interventions with outcomes	Page 2, line 53
Clinical Findings	6	Describe significant physical examination (PE) and important clinical findings.	Page 2, line 58
Timeline	7	Historical and current information from this episode of care organized as a timeline	Page 2, line 71
Diagnostic Assessment	8a	Diagnostic testing (such as PE, laboratory testing, imaging, surveys).	Page 2, line 65
	8b	Diagnostic challenges (such as access to testing, financial, or cultural)	Page 2, line 71
	8c	Diagnosis (including other diagnoses considered)	Page 3, line 83 and 86
	8d	Prognosis (such as staging in oncology) where applicable	Page 3, line 98
Therapeutic Intervention	9a	Types of therapeutic intervention (such as pharmacologic, surgical, preventive, self-care)	Page 3, line 88
	9b	Administration of therapeutic intervention (such as dosage, strength, duration)	Page 3, line 90
	9c	Changes in therapeutic intervention (with rationale)	Page 3, line 96
Follow-up and Outcomes	10a	Clinician and patient-assessed outcomes (if available)	Page 3, line 91 and 98
	10b	Important follow-up diagnostic and other test results	not applicable
	10c	Intervention adherence and tolerability (How was this assessed?)	not applicable
	10d	Adverse and unanticipated events	not applicable
Discussion	11a	A scientific discussion of the strengths AND limitations associated with this case report	Page 4, line 162 and 173; Page 5, line 198
	11b	Discussion of the relevant medical literature **with references**.	Page 3, line 101
	11c	The scientific rationale for any conclusions (including assessment of possible causes)	Page 4, line 176
	11d	The primary "take-away" lessons of this case report (without references) in a one paragraph conclusion	Page 5, line 206
Patient Perspective	12	The patient should share their perspective in one to two paragraphs on the treatment(s) they received	not applicable
Informed Consent	13	Did the patient give informed consent? Please provide if requested	Yes ☑ No ☐
